# Effects of Different Swimming Pool Conditions and Floor Types on Growth Performance and Footpad Dermatitis in Indoor-Reared White Roman Geese

**DOI:** 10.3390/ani11061705

**Published:** 2021-06-07

**Authors:** Shih-Chieh Liao, Pei-Xuan Lu, Shih-Yi Shen, Chih-Chang Hsiao, Ching-Yi Lien, Sheng-Der Wang, Tsung-Yi Lin, Po-An Tu

**Affiliations:** 1 Changhua Animal Propagation Station, Livestock Research Institute, Council of Agriculture, Changhua 512, Taiwan; ji3g4ike@mail.tlri.gov.tw (S.-C.L.); lu820101@mail.tlri.gov.tw (P.-X.L.); f40309@mail.tlri.gov.tw (S.-Y.S.); ccchang@mail.tlri.gov.tw (C.-C.H.); cylien@mail.tlri.gov.tw (C.-Y.L.); wsd@mail.tlri.gov.tw (S.-D.W.); 2 Hsinchu Branch, Livestock Research Institute, Council of Agriculture, Miaoli 36848, Taiwan; tylin@mail.tlri.gov.tw

**Keywords:** White Roman geese, indoor rearing, swimming pool conditions, floor types, footpad dermatitis

## Abstract

**Simple Summary:**

The goose industry provides meat and down to the food and textile processing industry and is the third-largest poultry industry in Taiwan after the chicken and duck industries. After the avian influenza virus (HPAIV) pandemic in 2015, most poultry farms in Taiwan have been restricted to closed birdhouses to improve biosafety. However, indoor-raised poultry may experience footpad dermatitis problems. We studied the effects of providing a swimming pool and different floor types on the growth performance and footpad dermatitis score for indoor-reared White Roman geese to reduce the risk of footpad dermatitis. Our data indicated that the incidence of footpad dermatitis was decreased during the feeding period in geese supplied with a swimming pool. Our findings may help improve animal welfare in modern waterfowl production by having the geese express their natural behaviors with water.

**Abstract:**

Footpad dermatitis (FPD) is a major foot disease in modern poultry production, and it affects both poultry health and animal welfare. It refers to inflammation and necrotizing lesions on the plantar surface of the footpads and toes. We investigated the effects of providing a swimming pool and different floor types on growth performance and FPD score in indoor-reared White Roman geese. Forty-eight male and 48 female White Roman geese were randomly allocated to pens with or without a swimming pool and with either mud or perforated plastic floor and reared from 15 to 84 days of age. Growth performance measurements included feed intake (FI), weight gain (WG), and feed conversion ratio (FCR). FI, WG, and FCR were significantly decreased at various growth periods in geese provided with a pool. Lower WG and bodyweight for the perforated plastic floor group were found at 15–28 and 28 days of age, respectively. The geese reared on the perforated plastic floors without a pool had higher FPD scores at 70 and 84 days of age than those with other rearing conditions. A higher incidence of FPD score 1 was observed in geese raised without a pool. In conclusion, providing a pool can improve footpad health in indoor-reared White Roman geese but may not benefit growth performance.

## 1. Introduction

The goose industry has been steadily developing and provides meat and down to the food and textile processing industry. It is the third-largest poultry industry in Taiwan, after the chicken and duck industries. White Roman goose is the most popular breed, accounting for 97% of the breeding goose population in Taiwan. Most traditional goose farms in Taiwan use free-range rearing systems. This system has a high biosecurity risk because the goose can easily come in contact with wild birds. Since 2014, new subtypes of highly pathogenic avian influenza virus (HPAIV) that have descended from H5 viruses have emerged and spread rapidly worldwide through the migratory bird flyways [1,2]. The new clade of HPAIV has caused major death and culling of infected birds in poultry farms. In 2015, three new subtypes—H5N2, H5N3, and H5N8—caused a severe epidemic in Taiwan [3]. The infection had high mortality; the infected geese died quickly, and more than 1.4 million geese were culled. This epidemic severely affected the goose industry in Taiwan, and the relative output value also plummeted by 64.2% compared with that in 2014. The Taiwanese government revised the law that mandatory closed birdhouses and facilities should be established to improve biosecurity. Therefore, the goose industry has an increasing demand for closed goose houses for indoor rearing. According to recommendations concerning domestic geese of the Standing Committee of the European Convention, the environment and management have to fulfill the animal’s biological requirements rather than "adapt" the animals to the environment through mutilations [4]. However, the uncertainty of cost-effectiveness and return on investment reduces the willingness of farmers to invest in concrete housing for geese rearing. Hence, during the transition from free-range rearing to indoor rearing, some animal welfare problems such as footpad dermatitis (FPD) and feather pecking caused by the environment and management must be addressed.

FPD is a major foot disease affecting poultry health and animal welfare in modern poultry production. The main symptoms of FPD include hyperkeratosis, scaly lesions, abrasion, ulcers, swelling, cracking, bleeding, inflammation, and tissue necrosis in the footpads, which is most likely to induce pain and inconvenience to the birds, hindering their rest and natural behaviors [5,6,7,8,9,10]. In addition, the severely ill birds may reduce their drinking and eating habits [6], which is more likely to cause weakness and death. FPD is also known as pododermatitis or contact dermatitis. All these terms generally refer to plantar skin diseases characterized by superficial-to-deep inflammation and necrotizing lesions on the plantar surface of the footpads and toes [6]. Deep tissue ulcers can cause abscesses and thickening of the subcutaneous tissue structure [6]. In addition, a bacterial infection of the plantar skin wound can easily cause gangrenous dermatitis. Hock burns and breast blisters are rarely caused by bacterial infections but are often considered contact dermatitis [6]. Therefore, the footpads, hock joints, chest injuries, and FPD incidence in poultry farms have been used as vital animal welfare indicators in Europe [6,10].

The quality and moisture content of litter are key factors affecting FPD [6,10,11,12,13]. Other factors include the depth and material of litter, stocking density, environmental temperature and humidity, drinking water facilities, management, sex, body weight, genetics, nutrients, feed quality, and incubation temperature [6,10,11,14,15,16]. For FPD prevention, studies have focused on improving the quality and management of litter [12,13]. From the animal nutrition and physiology perspectives, the main strategies adopted have been increasing the nutrient content and utilization of feed and reducing the quantity or changing quality of feces to maintain good litter quality, thereby improving the animals’ skin structure and immunity [7,10,17,18,19,20,21,22]. In addition, some poultry farms have begun to use full mesh floors [23]. Studies have also investigated the effect of floor material and design on FPD [16,24,25,26,27,28]. However, the results were not consistent between different poultry species (chicken, turkey, and duck) or even in the same species (chicken). This may require further investigation of the material, wire diameter, mesh size, surface design (smooth or rough) of the floor used, and the mechanical pressure on the foot caused by the animal’s body weight.

Moreover, waterfowls such as ducks and geese may benefit from being provided a swimming pool compared with land birds. The water could be used to express their normal behavior and fulfill their biological requirements, especially the grooming ritual [4,29]. Other characteristics such as feather quality, feather cleanliness, eye cleanliness, nostril cleanliness, gait score, FPD, and other body condition score indicators have also been improved by providing a swimming pool [30,31,32,33,34,35].

The domestic goose industry has frequently informed us of feather pecking and foot health issues using a closed goose house in Taiwan. However, an official research project that investigated the foot health of 14 commercial goose farms in Taiwan revealed that severe FPD (more than 50% of geese) was common in geese production in 2017 (Table S1). This may be related to the ground material and design of the goose farm and other facilities. This study evaluated the effects of different swimming pool conditions and floor types on growth performance and FPD for indoor-reared White Roman geese to alleviate FPD in indoor-reared geese. Our findings may help establish the appropriate goose house conditions for reducing the FPD incidence during the feeding period.

## 2. Materials and Methods

### 2.1. Experimental Design

The White Roman geese used in this study were produced and maintained by Changhua Animal Propagation Station, Livestock Research Institute, Council of Agriculture, Executive Yuan, Taiwan. The experiments were performed following Taiwan regulations and approved by the Institutional Animal Care and Use Committee of Changhua Animal Propagation Station (approval number: 10806). For estimating the required sample size, we used the standard Type I error α = 0.05 and Type II error β = 0.2. The standardized effect size (d = 0.35) was calculated by the mean and standard deviation from our previous investigation. The nondirectional null hypothesis was considered a no-difference (Ho: μ1 = μ2) with the two-tailed distribution. Using the G*Power software [36], the required sample size was n = 94; then, we used 96 animals to detect a treatment difference.

### 2.2. Animal Housing

**Figure 1:**
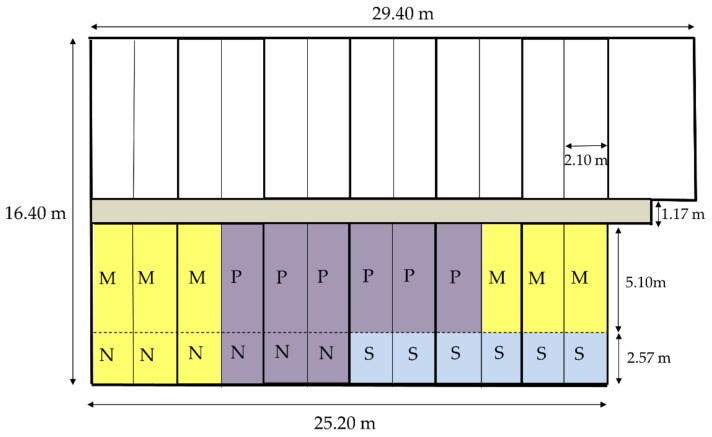
Planimetric map of experimental geese house. N: no swimming pool. S: swimming pool. M: mud floor. P: perforated plastic floor.

One-day-old goslings were raised to 14 days of age on stainless steel with a mesh floor (mesh size: 1 × 1 cm^2^, wire diameter: 0.1 cm) in closed goose houses. A round hole plastic soft net (mesh diameter: 1 cm, wire diameter: 0.2 cm) was paved on the floor to protect the geese’s footpad. The geese had ad libitum access to feed and drinking water, with 24 and 12 h of light during 1–14 and 15–84 days of age, respectively. The stocking density was 1.92 birds/m^2^. The geese were fed a starter diet containing 20% crude protein and metabolizable energy of 2900 kcal/kg from 0 to 28 d and a grower diet containing 15% crude protein and metabolizable energy of 2800 kcal/kg from 29 to 84 days of age (Table S2). At 15 days of age, the geese were moved to and raised in a closed goose house, an open-sided goose house surrounded by a plastic antibird net until the end of the experiment (12 weeks of age). The floor of the goose house was cemented and contained 24 pens. Each pen was separated by stainless steel and galvanized net. The dimension and area of the pen were 7.67 m × 2.10 m and 16.11 m^2^, respectively. Each pen consisted of a 5.10 m × 2.10 m cement ground and a 38-cm-deep swimming pool (dimensions: 2.57 m × 2.10 m). Only 12 pens on the side of the goose house were used in this study (Figure 1). The space of the cement ground was reduced to 5.10 m × 1.05 m to meet the stocking density of commercial geese management practice; because if the area was decreased to 10.75 m^2^ (5.10 m × 1.05 m + 2.57 m × 2.10 m), the stocking density could be maintained at 0.74 birds/m^2^ throughout the experiment. The experiment was performed during the hot season from June to August 2019 in Changhua County (23°51′32.2′′ N 120°33′29.6′′ E), and the environmental temperature was 23.16 to 37.66 °C in the goose house. Natural ventilation was used in the goose house. The swimming pool was emptied, washed, and refilled on Mondays and Thursdays. An 8-cm water depth (knee height for gosling) was supplied for the birds at 15 days of age and was gradually increased to 38 cm. All geese had ad libitum access to clean water and a pelleted grower diet. No additional washing and cleaning was applied to the goose house throughout the raising period to mimic a commercial goose farm’s management model.

### 2.3. Experimental Design and Growth Performance

**Figure 2:**
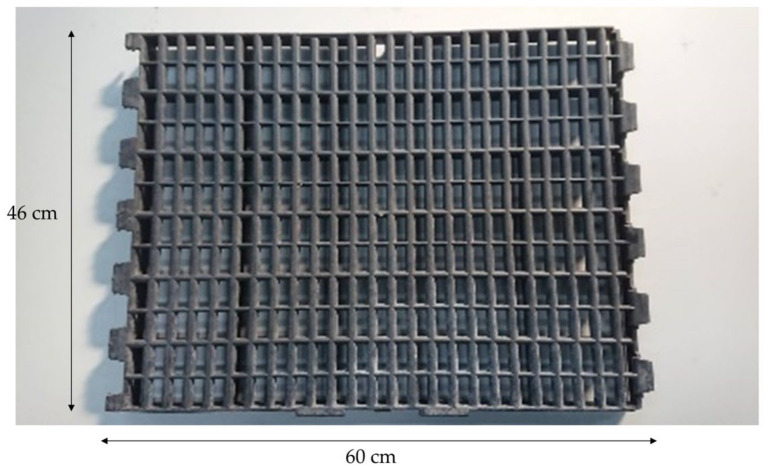
Perforated plastic floor used in the experiment. The size of each perforated plastic floor was 46 × 60 cm. The mesh size was 1 × 3 cm and the wire diameter was 1.2 cm.

Ninety-six White Roman geese (48 males and 48 females) were randomly divided into a factorial design of two swimming pool conditions and two floor types at 15 days of age. Each treatment was allotted to three pens with eight geese per pen. The swimming pool conditions were supplied with swimming pool (S) or not (N), whereas the floor types were mud floor (M) or perforated plastic floor (P). For the treatment of M and P, the 8-cm-thick, clean earth and 30-cm-high perforated plastic floor were installed on the existing cemented floor in the house, respectively. For N × M and N × P treatment, the swimming pool space was covered with the earth and perforated plastic floor, respectively. The perforated plastic floor (mesh size: 1 × 3 cm, wire diameter: 1.2 cm) used in this experiment is shown in Figure 2. No additional litter material was used. All the geese’s body weight was measured and averaged per pen at 15, 28, 56, and 84 days of age, respectively. The feed intake (FI) of geese was measured per pen. FI, weight gain (WG), and feed conversion ratio (FCR) were then calculated for 15–28, 29–56, 57–84, and 15–84 days of age.

### 2.4. FPD Scoring

The FPD scoring standards for geese were modified from the scoring system in ducks reported previously [7,32,37]. At 28, 42, 56, 70, and 84 days of age, FPD scores of geese within each pen were measured based on the criteria defined in Table 1. The footpads of both feet were cleaned before scoring. The proportion of FPD score level was calculated additionally as score 0 level (the score 0 to < 0.5), score 1 level (the score 0.5 to < 1.5), and score 2 level (the score 1.5 to < 2.0). The mean FPD score and the proportion of each score level were calculated per pen per treatment.

### 2.5. Statistical Analysis

The effects of swimming pool conditions and floor types and their interaction were assessed using the GLM procedure of SAS 9.4 [38]. Mean values and standard error of the mean (SE) were calculated for all parameters. FI, WG, and FCR were estimated at the pen level, as was the FPD score at 28, 42, 56, 70, and 84 days of age. The FPD score was evaluated using the mean of both feet and square-root transformed before analysis to satisfy the ANOVA requirements for normality and homogeneity of variance. The least-square means for FPD score were rerun using original pen level data, whereas *p* values were calculated using the transformed data with the same model. *p* < 0.05 was considered significant. Tukey’s post hoc test was used for separating the means.

The FPD score division of each pool condition × floor type treatment at 28, 42, 56, 70, and 84 days of age was analyzed using a proportional odds logistic regression model for ordinal responses using the PROC LOGISTIC procedure in SAS 9.4.

## 3. Results

### 3.1. Growth Performance

**Figure 3:**
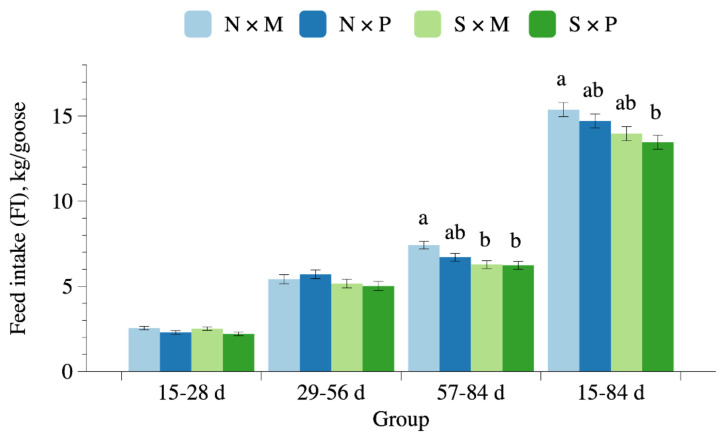
Feed intake in White Roman geese subjected to different floor types and pool conditions at different days of age. N × M: no swimming pool on the mud floor. N × P: no swimming pool on perforated plastic floor. S × M: swimming pool on the mud floor. S × P: swimming pool on perforated plastic floor. ^a^^,b^ Means with different superscripts within each group differ significantly (*p* < 0.05).

The growth performance data are presented in Table 2. Significant interactions of floor type and pool condition were found for FI at 57–84 and 15–84 days of age and for body weight at 56 days of age (*p* < 0.05). Geese reared in N × M pens had higher FI at 15–84 days of age than those reared in S × P pens (*p* < 0.05; Figure 3). At 57–84 days of age, geese reared in S*M and S*P pens had lower FI than those reared in N × M pens (*p* < 0.05). Furthermore, geese receiving the N treatment exhibited higher body weight at 56 days of age, WG at 29–56 days of age, FI at 57–84 and 15–84 days of age, and FCR at 15–28 days of age than those receiving the S treatment (*p* < 0.05). Geese receiving the M treatment exhibited higher body weight at 28 days of age and WG at 15–28 days of age than those receiving the P treatment (*p* < 0.05).

### 3.2. FPD

**Figure 4:**
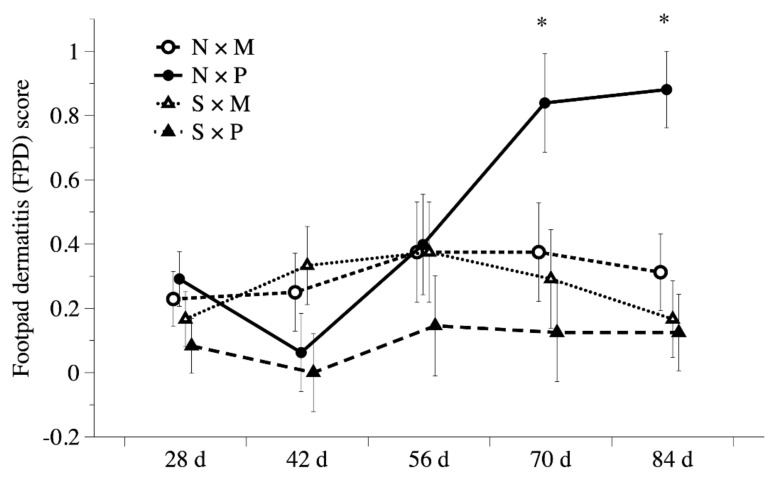
Comparisons of FPD score in White Roman geese subjected to different floor types and pool conditions at different days of age. N × M: no swimming pool on the mud floor. N × P: no swimming pool on perforated plastic floor. S × M: swimming pool on the mud floor. S × P: swimming pool on perforated plastic floor. * *p* < 0.05.

**Figure 5:**
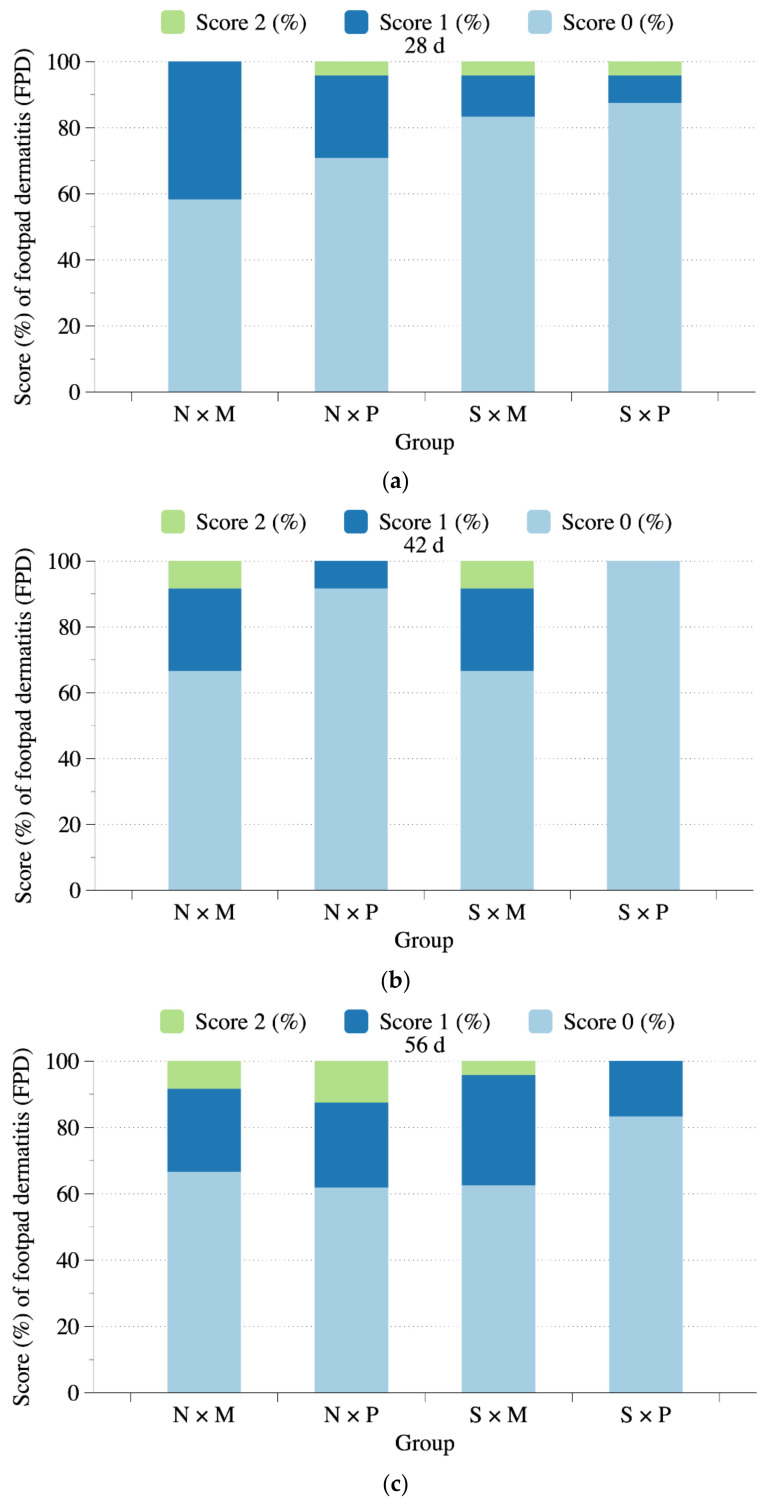

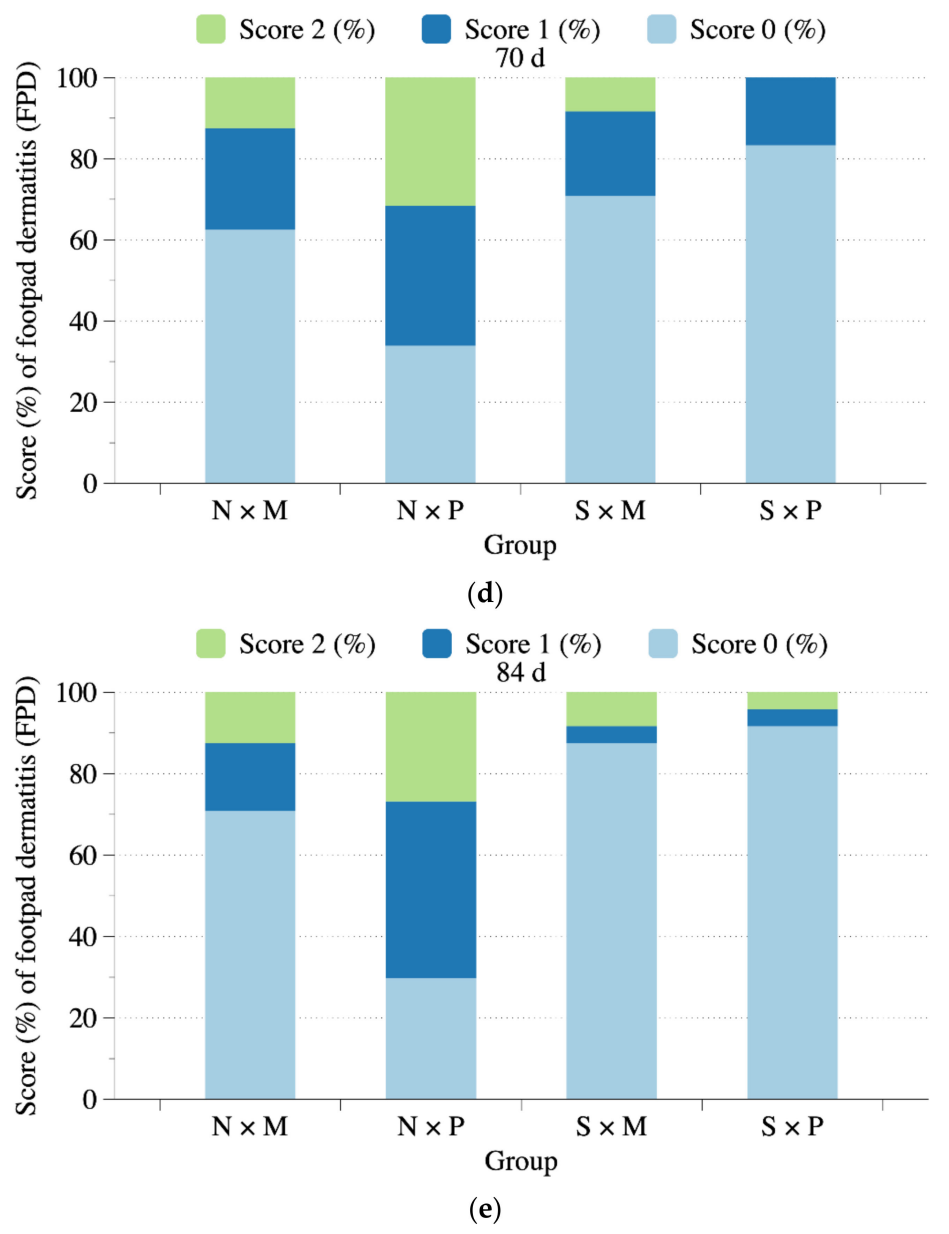
FPD score levels division (%) per treatment combination at 28 (**a**), 42 (**b**), 56 (**c**), 70 (**d**), and 84 (**e**) days of age as score 0 level (the score 0 to <0.5), score 1 level (the score 0.5 to <1.5), and score 2 level (the score 1.5 to <2.0). N × M: no swimming pool on the mud floor. N × P: no swimming pool on perforated plastic floor. S × M: swimming pool on the mud floor. S × P: swimming pool on the perforated plastic floor.

Figure 4 presents the results of the FPD score in White Roman geese subjected to different floor types and pool conditions. At 70 and 84 days of age, interactions of the pool conditions and floor types were significant (*p* < 0.05). Among all pen conditions, the geese reared in N × P pens had the worst FPD score at 70 and 84 days of age (*p* < 0.05). Higher FPD scores were observed in geese subjected to N and P treatments (*p* < 0.05). No difference in the FPD scores was observed among the groups before 56 days of age. Moreover, significant interactions of the swimming pool conditions and floor types were detected on the distribution over the FPD scores at 84 days. The N × P pens showed the highest incidence of FPD score 1 and had a lower incidence of FPD score 0 than S × M and S × P pens at 84 days of age (Figure 5). Furthermore, geese receiving the N treatment had a higher incidence of FPD score 1 and a lower incidence of FPD score 0 than those receiving the S treatment at 84 days of age (*p* < 0.05). Geese receiving the M treatment exhibited a lower incidence of FPD score 1, and a higher incidence of FPD score 0 than those receiving the P treatment at 84 days of age (*p* < 0.05).

## 4. Discussion

Compared with other poultry species, geese have some advantages such as greater tolerance to diseases and cold weather, less demand for equipped houses, and excellent fiber digestion ability [39,40]. Therefore, geese have been long raised by grazing or free-range rearing systems in outdoor areas, which were usually equipped with swimming pools, even in modern intensive farming [15,39,41]. Being waterfowl like ducks, geese are strongly water-oriented and require access to water for swimming and bathing to fulfill their biological needs [4,29]. However, the outdoor swimming pool poses a considerable biosecurity risk for interaction with wild birds, whose excrement fell into the pool [41]. According to animal welfare considerations and waterfowl literature [4,29,30,31,32,33,34,35], water bath facilities are essential for geese. Even in the goose house, it is recommended to provide a sufficient water bath facility [4]. However, providing an indoor pool to indoor-reared geese may also be detrimental to biosafety and water conservation. The geese are more likely to touch or drink each other’s feces and contract diseases. Although animal welfare is very important, it must be operated on the premise of considering biosafety and farmers’ economic benefits. This requires more research works to find suitable solutions. In our study, when geese were raised in pens equipped with a swimming pool, they exhibited lower body weight, WG, FI, and FCR. Chen et al. [30] also reported that providing water baths or forced water baths on high-bed strips reduced the WG and FI of White Roman geese at 4 weeks of age, especially in the forced water bath group. They speculated that the decreased FI might be due to the hot weather, which prompted geese to play in the water and spend more time bathing and feather finishing than feeding. By contrast, ducks provided with an indoor swimming pool exhibited improved growth performance [34,35]. This may be due to interspecific and environmental differences.

FPD has always been an essential indicator of animal welfare in commercial poultry farms. Controlling the quality and water content of litter for poultry indoor housing systems effectively prevents FPD [12,13]. Commercial farms for broilers, turkeys, and ducks generally place litter over the concrete floor. Using the concrete floor for poultry rearing is beneficial for all-in/all-out management and sanitizing. However, most geese houses still use litter on mud floors; litter contaminated by feces increases FPD risk because animal feet can contact feces-deteriorated litter. In addition, the cleaning and processing of used litter—whether disposing or recycling—generate additional costs. Hence, floor-raised geese are more likely to exhibit better growth performance and health than those raised on the mud floor, making the additional investment worthwhile. In our study, lower body weight at 28 days and WG at 21–28 days were observed in geese raised on perforated plastic floors, but the differences were not significant at 56 and 84 days of age. These results were slightly different from previous studies, which reported improved body weight or WG in broilers [24,25,26,27]. Liu et al. [42] reported that Yangzhou geese raised on a wire floor had higher body weight, WG, and FCR.

Similarly, a higher average daily WG of Peking duck rearing on plastic slatted floors than pine shaving litter was found [43]. By contrast, Lin et al. [44] observed that White Roman geese raised on cement floors had higher body weight at 84 days of age than those raised on cement slated floors. Cherry valley ducks raised on plastic nets had lower daily WG and FCR than those reared on sawdust bedding [45]. De Almeida et al. [24] and Almeida et al. [25] reported that the perforated plastic floor may damage broilers’ motility compared with the wood chip floor. Although the environment of the present study was considerably different from that employed by Lin et al. [44], similar undesirable results were obtained. This was probably caused by hindered bird activity by relatively large mesh holes on the raised floor, which could not support young geese’s feet. Skeletal conformation was stabilized after the growth period (29–56 days of age) of geese, and body weight was similar between the P and M groups after 56 days of age. The interaction between the swimming pool and floor type suggested that FI between the two P groups was not influenced by providing a swimming pool at 57–84 days of age. We assumed that the better ventilation and cooling effect of raised-floor and swimming pool design could reduce heat stress to geese. Lower body weight at 56 days of age was found in geese raised in S × P pens. We assumed that high environmental temperature might cause geese to stay in the swimming pool more often, reducing the FI. Young geese were also more likely to have difficulty reaching feed and water on raised floors because of underdeveloped feet.

Commercial broilers, turkeys, and ducks are prone to developing FPD because most farms use litter in their intensive indoor system. Improper management deteriorates litter quality, which seriously affects animal welfare, reduces sanitary conditions, and increases the incidence of carcass injuries, resulting in considerable production losses. Geese are usually raised by grazing or in a free-range system, making them less susceptible to FPD. Still, the shift to indoor rearing may cause the aforementioned animal welfare problems. Higher FPD scores were observed in free-range Turkish geese [15] because of the deteriorated ground environment as the vegetation degraded. Severe FPD may reduce growth performance and carcass value. In particular, goose palm is a popular delicacy in East Asia. Our study revealed that the FPD score of geese greatly improved when the swimming pool was supplied. In contrast, a study on ducks found that higher FPD scores for providing 4 h than 2 h of outdoor activity in the swimming pool, probably caused by increased standing time in the swimming pool [35]. Farghly et al. [34] did not observe a significant difference in the FPD score for ducks with 2 h activity in an outdoor swimming pool. We did not limit geese access to swimming pools in this study. Further research is warranted to examine the effects of limiting pool activity on animal behaviors. We observed higher FPD scores for geese reared on perforated plastic floors at 70 and 84 days of age, which were different from the results in broilers and turkeys [16,26,27,28]. Fully slatted flooring did not affect the FPD score in chickens but improved that of turkeys [27]. Çavuşsoğlu and Petek [16] and Çavuşsoğlu et al. [26] observed lower FPD scores in chickens reared on slatted floor. Partially perforated flooring systems also significantly improved the FPD score for chickens [28]. Abdel-Hamid et al. [46] observed higher FPD scores and incidence in cage-fed Muscovy ducks than in those raised in the litter.

The FPD score levels also reflected the FPD incidence for geese at different ages. At 84 days of age, the S treatment significantly reduced the incidence of FPD score 1 and increased that of score 0, whereas the P treatment had a higher incidence of FPD score 1 than that of M treatment. These results were different from the study on Peking ducks by Karcher et al. [37], which stated that ducks raised on perforated plastic floors had a higher incidence of FPD score 0 than those raised on pine shaving litter. Çavuşsoğlu and Petek [16] and Çavuşsoğlu et al. [26] also observed that broiler chickens reared on slatted floor had a significantly lower incidence of FPD than those raised on litter. However, de Almeida et al. [24] and Almeida et al. [25] observed that broiler chickens raised on perforated plastic floors had a higher incidence of FPD score ≥ 2 than those raised on wood shaving litter. Chuppava et al. [27] discovered that the perforated plastic floors did not affect the FPD score distribution for broiler chickens but significantly improved it for turkeys. We noted that the feces were more likely to stick on the plastic floor than on the mud floor; thus, as the birds’ body weight increased, their footpads were more likely to contact feces stuck in the plastic mesh, thereby increasing the FPD score. By contrast, when the geese were provided with a pool, they tended to be more likely to clean the sticky feces during bathing, thus lowering FPD score and incidence.

In this study, providing a pool reduced the FPD score of geese considerably, as did the use of mud flooring. In addition to the significant interaction of the two treatments at 70 and 84 days of age, higher FPD scores were found in geese kept in pens with perforated plastic flooring and no pool (Figure 4). The FPD scores among groups were not significantly different before 56 days of age. The division of FPD score levels of geese for each group at each time point is summarized in Figure 5. It also showed the same trend that the cumulative treatment effect on the FPD for the geese became even more obvious from 56–84 days of age. The geese reared in N × P pens had the lowest percentage of FPD score 0. Among all four rearing combinations (S × P, S × M, N × P, and N × M), the N × M group had the lower FPD score 0 and higher FPD scores 1 and 2, although the differences were not significant before 84 days of age. This implies that the increase in body weight due to higher pressure on the footpad may aggravate FPD. The marketing age of White Roman geese is approximately 84 d in Taiwan, and the goose skeleton is fully developed around 56 days of age. Therefore, the treatment effects may become more obvious in the later growth stages. Da Costa et al. [11] also indicated that FPD scores were highly correlated with the body weight-induced mechanical pressure on paws in turkeys. Studies on broiler chickens and geese have noted similar cumulative treatment effects over time [15,16,28]. The negative effects were highly related to the body weight pressure on footpads with the increase in age. In this study, we observed that the feces on the footpad could be washed away with their natural water bathing activity in the swimming pool. The FPD incidence can be reduced in both mud and perforated plastic floors when provided with a pool, with better improvement with perforated plastic floors.

## 5. Conclusions

This study evidenced the improvement for footpad health in indoor-reared White Roman geese when provided with swimming pools; however, this may also cause adverse effects on growth performance. Future studies should evaluate the effects of different swimming pool designs or water bathing facilities for the advancement of both animal welfare and the production performance of indoor-reared geese. The design should aim to maximize animal welfare, water saving, waste reduction, and biosafety while minimizing the negative effects on growth performance in geese.

## Figures and Tables

**Table 1:** Criteria of footpad dermatitis score in geese.

Score	Definition	Clinical Case and Description	
0	Heel and toe pads with no or very small superficial lesions (<0.5 cm in diameter), slight discoloration, mild thickening or keratinizing of the skin.	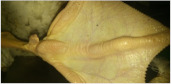	Normal geese foot.
1	Lesions cover less than 50% of the fins, heel and toe pad area. Fins, heel and toe pads have more than 0.5 cm superficial lesions, crack, keratinization, discoloration, dark papillae.	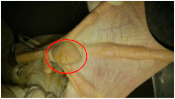	Toe pads have more than 0.5 cm cracks.
		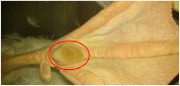	The papillae have a browning discoloration area.
2	Lesions or callouses cover 50% or more of heel and toe pads or any bloody lesions. Severe ulcers, scabs, bleeding, or swelling.	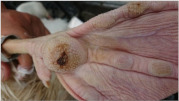	Clearly and severe ulcers on the toe pads with some discoloration area.
		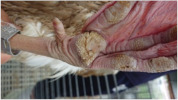	Keratinization’s cover more than 50% of the fins, heel and toe pad area.

**Table 2:** Effects of different floor types and pool conditions on growth performance in White Roman geese.

Experimental Groups	15–28 d of Age			29–56 d of Age			57–84 d of Age			15–84 d of Age			Body Weight		
	FI	WG	FCR	FI	WG	FCR	FI	WG	FCR	FI	WG	FCR	28 d	56 d	84 d
Water pool	NS	NS	*	NS	*	NS	**	NS	NS	*	NS	NS	NS	*	NS
N (n = 3)	2.42	1.20	2.00	5.563	1.75	3.17	7.06	0.94	7.61	15.04	3.90	3.85	1.98	3.73	4.68
S (n = 3)	2.36	1.13	2.09	5.092	1.56	3.25	6.25	0.94	6.79	13.71	3.65	3.75	1.88	3.44	4.39
Pooled SEM	0.07	0.03	0.03	0.185	0.05	0.06	0.16	0.07	0.47	0.29	0.09	0.05	0.04	0.07	0.10
Floor type	NS	*	NS	NS	NS	NS	NS	NS	NS	NS	NS	NS	*	NS	NS
M (n = 3)	2.53	1.25	2.01	5.292	1.67	3.16	6.85	0.92	7.50	14.67	3.85	3.81	2.02	3.69	4.62
P (n = 3)	2.25	1.08	2.08	5.363	1.64	3.26	6.47	0.97	6.90	14.09	3.70	3.80	1.84	3.48	4.45
Pooled SEM	0.07	0.03	0.03	0.185	0.05	0.06	0.16	0.07	0.47	0.29	0.09	0.05	0.04	0.07	0.10
Pool × floor	NS	NS	NS	NS	NS	NS	*	NS	NS	*	NS	NS	NS	*	NS

N = no swimming pool; S = with swimming pool; M = mud floor; P = perforated plastic floor; FI = feed intake, kg; WG = weight gain, kg.; CR = feed conversion ratio.; Pooled SEM = pooled standard error of means; NS = not significant at *p* > 0.05; * *p* < 0.05; ** *p* < 0.01.

## Data Availability

Data will be available from the first author upon request. The data are not publicly available due to institutional policy.

## Supplementary Materials

The following is available online at https://www.mdpi.com/article/10.3390/ani11061705/s1, Table S1. Investigation of footpad dermatitis data in Taiwan goose farms in 2017, Table S2: Composition of starter and grower diets.
