# Supplementary material for: Effects of Different Swimming Pool Conditions and Floor Types on Growth Performance and Footpad Dermatitis in Indoor-Reared White Roman Geese
Source: Animals (Basel). 2021 Jun 7;11(6):1705. doi: 10.3390/ani11061705 (PMC8228599; doi:10.3390/ani11061705)
Supplement: Supplementary file 1 [file animals-11-01705-s001.zip › animals-1221653-supplementary.pdf]

animals

MDPI

Table S1. Investigation of footpad dermatitis data in Taiwan goose farms in 2017.

Goose farm number	Location	Feeding scale (birds)	Floor condition	Outdoor playground	Outdoor water pool	Slaughter age (days)	Market weight (kg) [1]	Footpad dematitis score[2]	Incidence of footpad dermatitis (%)[3]
1	Yunlin County	300	Cement floor and muddy soil	yes	yes	96	5.4	0.1	10
2	Pingtung County	275	Stone ground	yes	yes	100	5.4	0.45	45
3	Chiayi County	540	Sandy soil	yes	yes	96	4.74	0.55	48
4	Yunlin County	400	Sandy soil	yes	yes	100	5.04	0.73	69
5	Pingtung County	1000	Stainless perforated floors and Stone ground	yes	yes	96	5.16	0.75	62
6	Chiayi County	550	Muddy soil	yes	yes	100	5.52	0.8	79
7	Pingtung County	100	Sandy soil	yes	yes	98	5.46	0.8	67
8	Chiayi County	125	Sandy soil	yes	yes	96	5.76	0.83	71
9	Pingtung County	350	Sandy soil	yes	no	99	5.1	1	86
10	Chiayi County	250	Sandy soil	yes	yes	101	5.22	1.01	67
11	Yunlin County	500	Muddy soil	yes	yes	96	5.22	1.12	89
12	Chiayi County	250	Sandy soil	yes	yes	100	5.4	1.18	93
13	Pingtung County	600	Stone ground	yes	yes	96	5.4	1.22	93
14	Yunlin County	150	Stone ground	yes	no	102	5.16	1.31	99

[1] The value represents the average weight of the feeding scale of geese. [2] Data sampled from 100 geese in each farm. [3] The value is calculated as the percentage of geese whose FPD score reaches 0.5 points or more.

animals MDPI

Table S2. Composition of starter and grower diets.

**Ingredients**	**Starter** (0 to 28 d)	**Grower** (29 to 84 d)
Yellow corn	61.60	64.20
Soybean meal	29.00	21.50
Wheat bran	-	5.00
Rice hull	-	3.00
Fish meal	3.50	-
Molasses	3.00	3.00
Salt	0.30	0.30
Dicalcium phosphate	1.30	1.60
Limestone, pulverized	0.70	0.80
Choline chloride, 50 %	0.10	0.10
DL-methionine	0.25	0.20
Vitamin premix[1]	0.10	0.10
Mineral premix[2]	0.15	0.15
Total	100	100
Calculated values		
Crude protein, %	20	15
ME, kcal/kg	2,900	2,800

[1] Each kilogram contained the following: Vitamin A, 10,000,000 IU; Vitamin D_3, 2,000,000 IU; Vitamin E; 20,000 g; Vitamin B_1, 2 g; Vitamin B_2, 5 g; Vitamin B_6, 3 g; Vitamin B_{12}, 0.03 g; Biotin, 0.2 g; Vitamin K_3, 3 g; D-calcium pantothenate, 10 g; Folic acid, 2 g; Nicotinic acid, 30 g.
[2] Each kilogram contained the following: Cu, 15.0 g; Fe, 100 g; Zn, 50 g; Mn, 80 g; Co, 0.25 g; I, 0.85 g; Se, 0.15 g.
